# 3D based on 2D: Calculating helix angles and stacking patterns using *forgi 2.0*, an RNA Python library centered on secondary structure elements.

**DOI:** 10.12688/f1000research.18458.2

**Published:** 2019-04-23

**Authors:** Bernhard C. Thiel, Irene K. Beckmann, Peter Kerpedjiev, Ivo L. Hofacker

**Affiliations:** 1 Department of Theoretical Chemistry, Faculty of Chemistry, University of Vienna, Vienna, 1090, Austria; 2 Department of Biomedical Informatics, Harvard Medical School, Boston, Massachusetts, 02115, USA; 3 Research Group Bioinformatics and Computational Biology, Faculty of Computer Science, University of Vienna, Vienna, 1090, Austria

**Keywords:** RNA, Python, RNA tertiary structure, RNA secondary structure, coaxial stacking, pseudo knots

## Abstract

We present *forgi*, a Python library to analyze the tertiary structure of RNA secondary structure elements. Our representation of an RNA molecule is centered on secondary structure elements (stems, bulges and loops). By fitting a cylinder to the helix axis, these elements are carried over into a coarse-grained 3D structure representation. Integration with Biopython allows for handling of all-atom 3D information. *forgi* can deal with a variety of file formats including dotbracket strings, PDB and MMCIF files. We can handle modified residues, missing residues, cofold and multifold structures as well as nucleotide numbers starting at arbitrary positions. We apply this library to the study of stacking helices in junctions and pseudoknots and investigate how far stacking helices in solved experimental structures can divert from coaxial geometries.

## Introduction

In RNA 3D structure prediction, knowledge-based potentials are commonly used, especially for coarse-grained approaches that are suitable for larger RNA molecules ^1^. The creation of such potentials requires knowledge extraction from solved RNA structures, usually taken from the Protein Data Bank (PDB) ^2^. PDB files and their newer replacement, MMCIF files, contain atomic coordinates and additional information in the header fields, but do not contain any base-pairing annotations. To extract information about base pairs and their types ^3^, dedicated software like MC-Annotate ^4^, RNAView ^5^, FR3D ^6^, DSSR ^7^ or the RNApdbee web server ^8^ is required. Due to the hierarchical organization of the RNA energy landscape ^9^, it is often most convenient to treat secondary and tertiary structure of RNA molecules separately and predict tertiary structures given a secondary structure ^10^.

For knowledge extraction from RNA-containing PDB files, it is highly desirable to have a software library at hand, which understands the semantics of RNA secondary structures and makes tasks like iterating over loops of a certain type straightforward. Ideally, such a library should be written in an easily accessible scripting language, be well documented and tested and should be available under an open-source license.

Libraries other than the forgi library presented here only partly fill these needs. The Vienna RNA package ^11^ can be used to predict secondary structures from sequence, including advanced features like G-Quadruplex prediction and incorporation of SHAPE data. It provides Python and Perl bindings, but deals exclusively with secondary structure. Biopython ^12, 13^ is useful for dealing with RNA sequence data and can be used to load RNA 3D structures, but has no dedicated support for RNA secondary structure. PyCogent ^14^, a library for genomic biology, has extensive support for nucleic acid sequences, but contains only a lightweight class for RNA secondary structures and code for pseudoknot removal ^15^. A stand-alone version of the latter was included into the forgi library under the terms of the GNU General Public License 3.0. More specialized libraries include modeRNA ^16^ (homology modeling, Python) and the FR3D suite ^6^ (RNA motif search, Matlab). Here, we present the forgi library ^17^, which is centered on RNA secondary structure elements (such as stems, bulges and loops) and makes them usable for 3D structure analysis. forgi aims at providing a high level API for many common operations, but can be easily extended with new functionality. The flexibility of providing an open source library in a scripting language is a clear benefit over other programs that are only distributed as binaries. While not restricted to work with PDB or MMCIF files, forgi shines especially where 3D information is analyzed in the context of its secondary structure environment.

## Methods

### Implementation

The forgi library ^17^ is strongly object-oriented, but takes advantage of module-level Python functions where appropriate. The core object representing the secondary structure is the BulgeGraph object, which holds a Sequence instance for the primary sequence. To include secondary structure based 3D coordinates, the BulgeGraph’s subclass, CoarseGrainRNA is available. For all-atom 3D analysis, the forgi library has a built-in integration with Biopython.

We will briefly describe the three main data structures that hold the sequence, secondary structure and the tertiary structure representation of an RNA.

***Primary structure: The Sequence class.*** Each BulgeGraph holds a Sequence object. Since forgi supports loading of data from PDB files (see below), this Sequence object has to account for many special cases arising from experimental considerations, which will be detailed in the following paragraphs. There are two numbering schemes commonly used for sequences: 1-based indexing and indexing based on an external reference. In particular, many sequences in structural experiments use “1” to dedicate the first residue of a biological macro-molecule, whereas the first residue actually used in the experiment can be upstream of the functional RNA (leading to negative indices) or after the start of the biological unit (leading to an index above 1). The latter is especially common if fragments of larger RNA molecules like the ribosome are studied. Finally, experimenters might decide to insert nucleotides in the middle of a molecule. In order not to affect the numbering of subsequent residues, these inserted residues get the same number as the previous one, followed by a letter (called insertion code).

To handle both kinds of indexing, the Sequence class distinguishes indices by type. Integer indices always refer to 1-based indexing, while tuples compatible to the indices used in Biopython’s PDB module are interpreted as the second kind of indices.

For many applications, it is necessary to restrict the RNA alphabet to 4 letters (i.e. 4 residue types), “G”, “C”, “A” and “U”. However, in the cell many RNA molecules are post-transcriptionally modified at certain positions. Many modifications, including the methylation of OH or NH _2_ groups and A to I editing, have been implicated with a variety of biological functions ^18^. During parsing of PDB files, we automatically convert such modified residues to the unmodified parent, but in addition store the modification as an annotation in the Sequence object. The corresponding unmodified parents for 3-letter codes of common modified residues were obtained from PDBeChem ^19^ ( http://www.ebi.ac.uk/pdbe-srv/pdbechem/) and the forgi library has the ability to query this database on the fly if it encounters a new 3-letter code.

Finally, many experimental 3D structures do not contain coordinates for all residues present in the experiment. The forgi Sequence class can store two version of the sequence, with and without missing residues, and the file parser can extract this information from PDB and MMCIF files.

**Figure 1.**
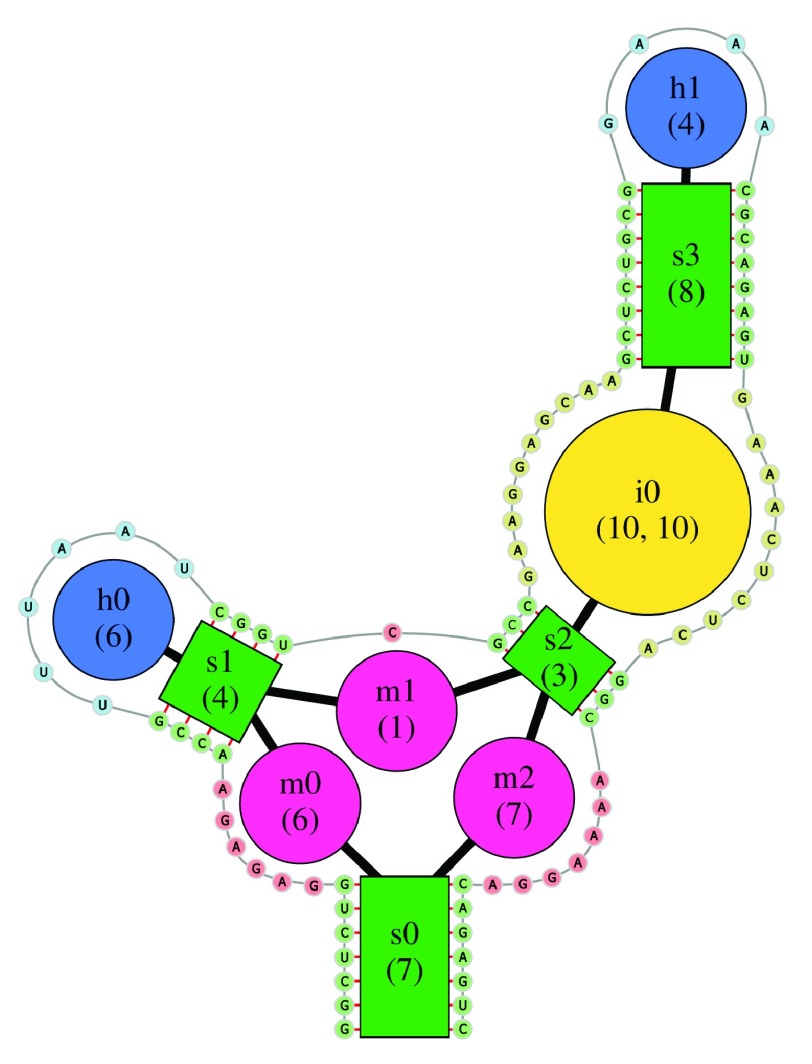
Illustration of the Bulge Graph representation underlying the forgi library. Secondary structure elements (stems, bulges and single-stranded regions) are nodes connected by edges. The sequence is shown around the Bulge Graph.

The secondary structure of an RNA is internally represented as a graph, the Bulge Graph, where secondary structure elements (stems, single-stranded regions, interior loops and hairpin loops) form the nodes. Whenever these elements are adjacent along the backbone, they are connected by an edge in this graph. During the Bulge Graph creation, each node gets a unique name such as “s0” for the first stem or “h0” for the first hairpin. The concept of the Bulge Graph, illustrated in Figure 1, has been described previously in more detail ^20^ and is related to the independently developed RAG (RNA as Graph) approach ^21^.

forgi supports element-based transformations of the Bulge Graph, such as condensing the secondary structure to an representation similar to RNAshapes ^22^, which we use to classify pseudoknots, for example (see below).

The BulgeGraph object allows for easy identification, selection and classification of structural domains, such as multi loops, helices consisting of multiple stems and bulges (termed “rods” in forgi), and pseudoknots.

***The CoarseGrainRNA class holds 3D structures.*** 3D structures are loaded from PDB or MMCIF files using Biopython’s PDB module ^13^. In order to assign a secondary structure to the RNA, forgi can call either MC-Annotate ^4^ (Linux only, no MMCIF-support, binary available at http://major.iric.ca/MajorLabEn/MC-Tools.html) or DSSR ^7^ (binary available after free registration at http://forum.x3dna.org). As an alternative, forgi has a built-in heuristic for the detection of canonical base pairs and GU wobble pairs. This heuristic is based on distances along the hydrogen bonds and the coplanarity of the bases, and is not intended to compete with the power of more specialized tools since it will fail in some edge cases, e.g. involving modified residues or residues with missing atoms. This heuristic is a useful fallback, if the above mentioned programs are not available.

During loading of the RNA, a helix axis is assigned to stems as described previously ^20^. For each stem, we store start and end coordinates of the helix axis as well as two twist vectors that point towards the minor groove at the beginning and the end of the stem. Similarly, start and end coordinates for bulges, loops and single-stranded regions are stored and can be accessed using the element’s name.

forgi 2.0 now fully supports co- and multi-fold structures and can load multiple chains that are connected by base pairs into a single CoarseGrainRNA object, while chains not connected by any base pair are loaded into separate objects.

Using Biopython’s KD-Tree implementation ( Bio.PDB.NeighborSearch), a list of residues within 6 angstrom from a non-RNA C or N atom is obtained. These residues are considered protein-/ligand interacting. Knowledge of interacting residues is particularly useful to avoid biases in statistics about structural features of bare RNA.

A cleaned version of the PDB - with modified residues converted to their canonical parent and non-RNA molecules removed is stored as Biopython chains in the CoarseGrainRNA’s chains attribute.

### Operation

forgi ^17^ is compatible with Python 2.7, 3.5 and 3.6, should run on all operating systems where its dependencies are available and has been tested on Linux, Mac and Windows. It makes heavy use of the NumPy ^23^ library to speed-up array-based calculations and also depends on SciPy ^24^, NetworkX ^25^, Biopython ^12, 13^, pandas ^26, 27^ and appdirs, all available via the Python Package Index (PyPi) or Anaconda.

***Helpful utility scripts.*** forgi comes with the two very useful scripts: rnaConvert.py can be used to convert between many common file formats for RNA structures, including the Vienna format and fasta variants, the bpseq format, the connectivity table (ct) format, MMCIF format and PDB files. visualize_rna.py can be used to display a coarse-grained representation of an RNA’s secondary structure in PyMol alongside the all atom structure from PDB files, producing visualizations like those in Figure 3 and Figure 5.

***Analysis of stacking geometries.*** To illustrate how the forgi library makes secondary structure elements usable for 3D structure analysis, we used it to analyze the stacking of adjacent helices in multi loops and pseudoknots in a representative set of RNA 3D structures ^28^ (version 3.36, available at http://rna.bgsu.edu/rna3dhub/nrlist/).

While loading these 3D structures into forgi, DSSR ^7^ (Version v1.7.1-2017nov01) was called to obtain the secondary structure and nucleotide level reference stacking annotations. We count a pair of connected helices as stacking, if at least one nucleotide of the first helix’s closing/opening base pair is in a continuous stack with at least one nucleotide of the second helix’s opening/closing base pair. This allows for any number of stacking nucleotides between the stems (not necessarily connected via the backbone) and for bulged out nucleotides which do not contribute to the stack. Our definition of stacking is more relaxed than stricter criteria used elsewhere ^29^.

For each pair of adjacent stems within a junction, we used forgi to calculate a number of properties, such as the angle between the stem vectors, the separation vector between the stems’ ends and an offset value between the stems.

These properties are based on the axes that were fitted to the helices (see above) and the multi loop segment that connects the two stems. We define the stem vectors ***v*** *_h_* as pointing away from the multi loop segment along the helix axis, where ***s*** *_h_* and ***e*** *_h_* are the helix’s start and end coordinates.

$$v_{h}\;=\;\;\left\{ \begin{array}{l} e_{h}\;–\;\;s_{h}\;\;\;\;\;\text{if}\;\;s_{h}\;\;\text{is}\;\;\text{at}\;\;\text{the}\;\;\text{side}\;\;\text{of}\;\;\text{the}\;\;\text{multiloop}\\ s_{h}\;–\;\;e_{h}\;\;\;\;\;\text{if}\;\;e_{h}\;\text{is}\;\;\text{at}\;\;\text{the}\;\;\text{side}\;\;\text{of}\;\;\text{the}\;\;\text{multiloop} \end{array}\right.$$

The angle between the adjacent stems *i* and *h* is then defined as the angle between their stem vectors ***v*** *_i_* and ***v*** *_h_*:

$$\text{cos}(\alpha )\;=\;\frac{v_{h}\cdot v_{i}}{\left\|v_{h}\right\|\cdot \left\|v_{i}\right\|}$$

The offset between stems is calculated as distance between two rays *R _h_* that start at the helix’s end closer to the multi loop segment, ***c*** *_h_*, and extend the helix axis:

$$c_{h}\;=\;\;\left\{ \begin{array}{l} s_{h}\;\;\;\;\;\text{if}\;\;s_{h}\;\;\text{is}\;\;\text{at}\;\;\text{the}\;\;\text{side}\;\;\text{of}\;\;\text{the}\;\;\text{multiloop}\\ e_{h}\;\;\;\;\;\text{if}\;\;e_{h}\;\text{is}\;\;\text{at}\;\;\text{the}\;\;\text{side}\;\;\text{of}\;\;\text{the}\;\;\text{multiloop} \end{array}\right.$$

$$R_{h}\;=\;\left\{X|X\;=\;c_{h}\;+\;\lambda v_{h},\;\lambda \;\;\geq \;0\right\}$$

Depending on the helix orientation, the distance between these rays is either the distance between two lines or the distance between two points or between one point and one line, all of which can be calculated with standard formulas.

The following code shows how forgi was used to calculate these properties for multi loops:

from forgi import load_rna
from forgi.threedee.utilities.vector import vec_angle, vec_distance
from forgi.threedee.utilities.vector import line_segment_distance

def main():
    # Load the PDB into CoarseGrainRNA instances
    # rnas is a list, because a PDB can contain multiple connected components 
    # This also loads the DSSR json annotations.
    rnas = load_rna("path/to/file.cif", pdb_remove_pk=False,
                    pdb_annotation_tool="DSSR")
    for rna in rnas:
        for junction in rna.junctions:
             print_junction_parameters(rna, junction)
          

def print_junction_parameters(rna, junction):
    """
    Prints the angles and offsets of a junction.
    :param rna: A BulgeGraph Object
    :param junction: A list of element names (strings)
    """
    for ml in junction:
        # rna.edges holds adjacent nodes in the BulgeGraph
        stem1, stem2 = rna.edges[ml]
        # rna.coords holds the 3D coordinates of structure elements
        # rna.coords["s0"] returns a tuple start-, end-coordinates of stem "s0"
        # Furthermore rna.coords has the get_direction function to give the
        # vector pointing from the start to the end coordinate.
        direction1 = rna.coords.get_direction(stem1)
        direction2 = rna.coords.get_direction(stem2)
        # Get the indices into rna.coords for the stem sides closer to
        # and further away from the multiloop segment ml
        c1, f1 = rna.get_sides(stem1, ml)
        c2, f2 = rna.get_sides(stem2, ml)
        # Make sure the stem vector points away from the multiloop
        if c1 == 1:
            direction1 = - direction1
        if c2 == 1:
            direction2 = - direction2
        angle = vec_angle(direction1, direction2) # imported above
        is_stacking_dssr = (ml in rna.dssr.stacking_loops())
        closer1 = rna.coords[stem1][c1]
        closer2 = rna.coords[stem2][c2]
        # Calculate the offset as distance between rays.
        offset = vec_distance(*line_segment_distance(
                                  closer1, closer1+100000*direction1,
                                  closer2, closer2+100000*direction2))
        print("{}\t{}\t{}".format(angle, is_stacking_dssr, offset))

We then used pandas ^26, 27^, Matplotlib ^30^ and a custom library ( https://github.com/Bernhard10/filterAndView) to analyze and visualize the collected data. The results were collected for different classes of RNA independently. This was necessary, because the representative sets of RNA structures contain, by design, homologs of the same molecule in multiple species.

For the classification of pseudoknots, Reidys’ concept ^31^ based on the definition of the mathematical genus is used. Classes of pseudoknots are defined based on their shadow representations, which contain only crossing base pairs and only one base pair each. On the level of these shadows, only four distinct classes exhibit genus 1, two of which are well known: the H-type pseudoknot and the kissing hairpin. pseudoknots with higher genus contain, among others, the case where a genus 1 pseudoknot is nested within another pseudoknot.

In combination with the forgi library, we wrote a tool that is able to convert structures to their shadow representation, identify and classify the pseudoknots within the structure and describe their helix arrangement in the 3D structure. This tool, called pseudoknot_analyzer.py, is distributed with the forgi library in the folder “examples”. We used it to gather statistics about simple H-type and kissing hairpin pseudoknots. Figure 4a, b illustrates how we measured the angle between stems in pseudoknots via vector directions. In kissing hairpins the angles *α* and *β* restrict the possible values of the angle *γ*. We also include the representative structure of an intermolecular kissing hairpin interaction (lacking the green connection in Figure 4b) in our analysis. In this special case *α* and *β* are indistinguishable and were assigned arbitrarily.

### Results

**Figure 2.**
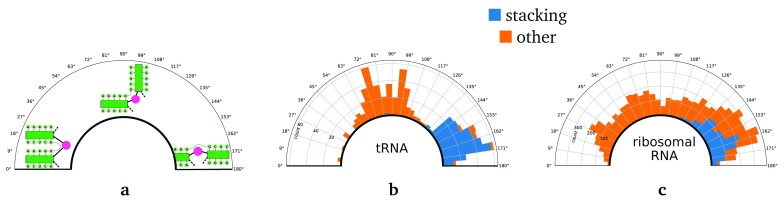
Distribution of angles between adjacent stems in multi loops. ( **a**) Angles close to 0° mean parallel stems whereas angles close to 180° correspond to potentially stacking stems. Angle distributions in ( **b**) tRNAs and ( **c**) ribosomal RNA are shown as a histogram mapped onto a circular plot illustrating the angles. The (inner) blue bars are instances where DSSR detects stacking on the atom-level scale. The orange bars start at the top of the blue bars and indicate geometries where DSSR does not detect stacking.

We used the helix-centered representation of the 3D structure to analyze the geometry of coaxially stacking helices. The relative geometry of two cylinders in 3D space can be described by five parameters: A separation vector (three parameters) and two angles for the relative orientation in 3D space. In Figure 2 and Figure 4, we show the single angle calculated between the vectors along the helix axes, as a proxy for these parameters.

**Figure 3.**
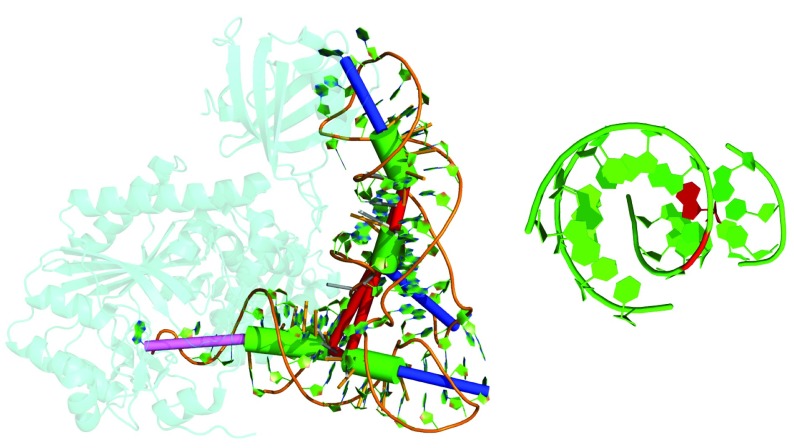
Example of a tRNA where stacking between two non-collinear stems occurs (PDB id 4WJ4 ^32^). Green cylinders were fitted to stems, blue cylinders represent hairpins and the pink cylinder the unpaired nucleotides at the 3’ end. Red cylinders connect stems and indicate single-stranded connections between these stems (multi loop segments). Left: The stems of the anticodon arm (top) and the D-arm stack (according to DSSR) despite being at an angle of 143°. Right: View along the axis of the anticodon arm’s stem. The red nucleotide is the unpaired multi loop segment which mediates stacking to the stem of the D-arm. This illustration was generated using PyMol ^33^ via the visualize_rna.py wrapper in forgi.

**Figure 4.**
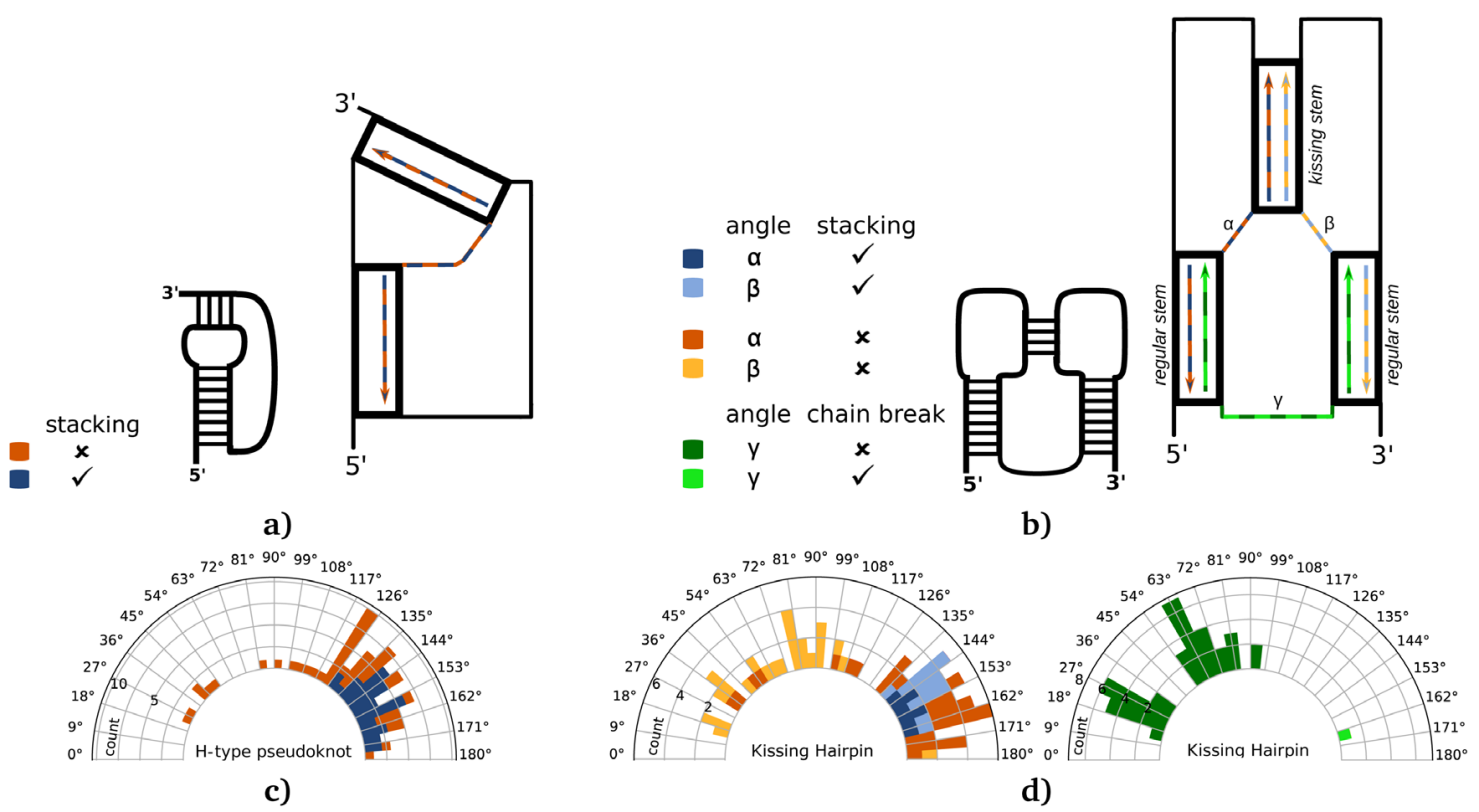
Angles between the stems in pseudoknots. ( **a**) and ( **b**) show the vector directions used for the angle measurement. The distributions of the angles between adjacent stems building **c**) an H-type pseudoknot or ( **d**) a kissing hairpin are shown as histograms mapped to a circle. Furthermore, the distribution of angles between the regular stems of kissing hairpin pseudoknots is shown in the second panel of ( **d**). Like mentioned in Figure 2, the angles in **c**) measured between stacking stems (detected via DSSR) are colored in blue. Geometries without stacking are colored in orange. The color scheme in ( **d**) (shades of orange, blue and green) refer to the respective vectors/measured angles (α, β, γ) in the same color scheme as in ( **b**). Blue bars stand for stacking between the two related stems, whereas orange ones for non-stacking stems. Dark green bars in the second kissing hairpin associated histogram show angles measured between a intramolecular interaction (kissing hairpin pseudoknot), whereas light green bars stand for angles measured between two RNA chains (kissing hairpin interaction).

**Figure 5.**
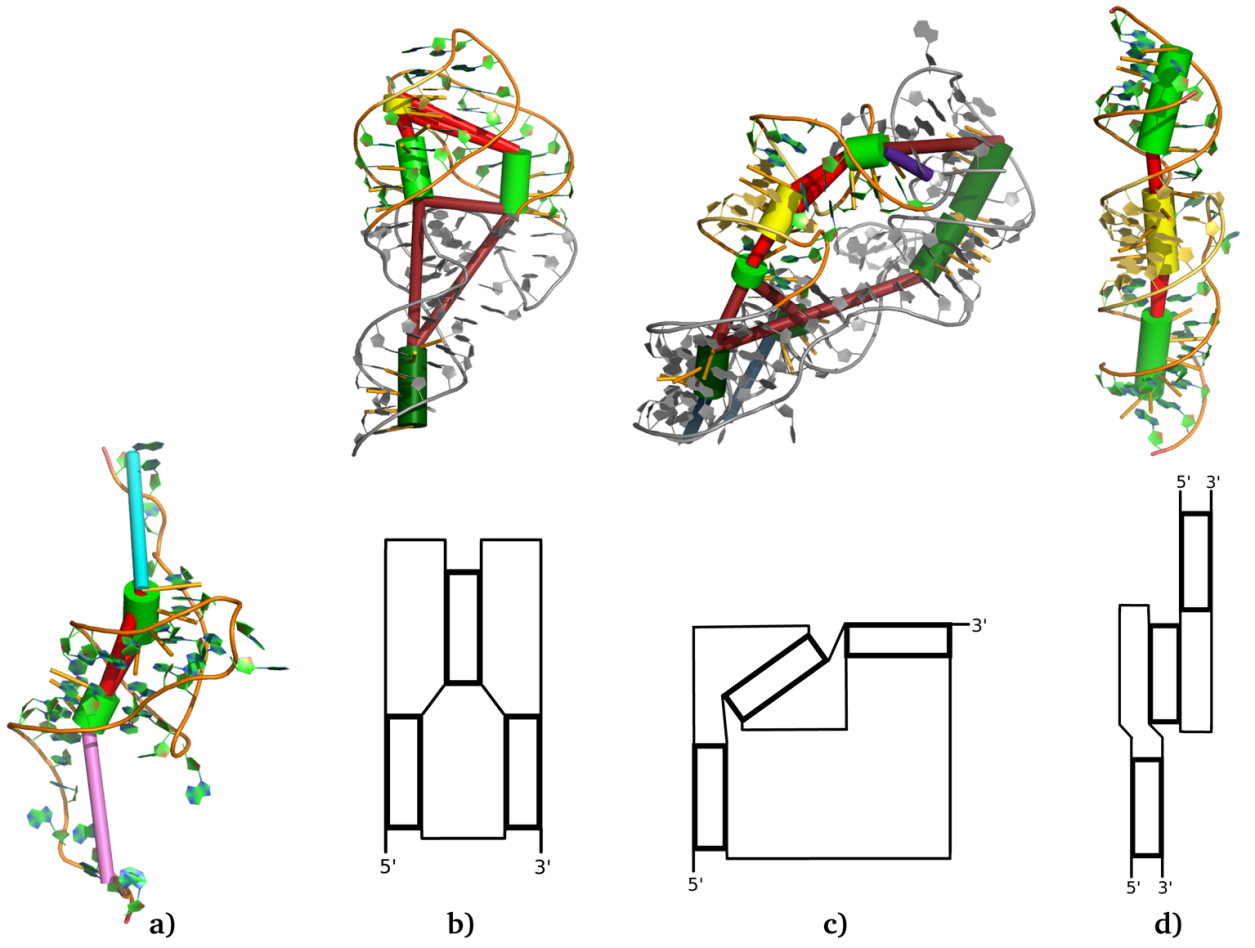
Examples of coaxial stacking within ( **a**) an H-type pseudoknot (PDB id 2XD0) and ( **b**- **d**) the three major structural families of kissing hairpins (PDB ids 5KPY, 4FRN and 1ZCI, from left to right). In all four representations the green cylinders were fitted to stems, the turquoise and pink cylinder represent the unpaired nucleotides at the 5’ and 3’ end respectively. Red cylinders connect stems and indicate single-stranded connections between these stems (pseudoknot or multi loop segments). The light-colored stems in ( **b**– **d**) represent the regular stems of kissing hairpins, whereas the kissing stem is colored yellow. Note that panel ( **d**) (PDB id 1ZCI) shows a kissing hairpin interaction between two RNA chains. Below the 3-dimensional representation of the kissing hairpins, we show a schematic sketch of the structural family’s helix arrangement. These illustrations were generated using PyMol ^33^ via the visualize_rna.py wrapper in forgi.

The distribution of angles between adjacent stems in tRNAs multi loops (see Figure 2a) shows the expected bimodal distribution with one mode slightly below 90° and another mode between 160° and 170°, which fits to the known helix arrangement in the L-shaped tRNA. As confirmed by comparison to annotations with DSSR, the second peak is almost exclusively due to stacking helices, even at angles as small as 140°. In Figure 3 we show an example of such a coaxial stack that has been strongly bent (possibly by the tRNA synthetase) without completely breaking the stack or affecting the canonical tRNA secondary structure.

The second family of RNA molecules with lots of data available is ribosomal RNA. Here we find that the angle of adjacent stems in multi loops is almost uniformly distributed above 20° until a peak from 135° to 170°, where DSSR annotates roughly half of the geometries as stacking (see Figure 2c). 7% of the stacking geometries and 67% of the non-stacking geometries with an angle above 140° also have an offset above 10Å between the extended stem axes.

Additionally we analyzed the angle distribution between the stems forming simple H-type pseudoknots or kissing hairpins. Most angles measured within an H-type pseudoknot are between 120° and 180° (see Figure 4c). In this range, 35 out of 67 instances correspond to stacking. One example within this range is shown in Figure 5a, which represents a processed non-coding RNA, which regulates a bacterial antiviral system (PDB id 2XD0 ^34^).

The distribution illustrated in Figure 4d shows mainly values between 130° and 180° for the angles *β* and especially *α*. One additional angle measurement between the two regular stems (see Figure 4d, angle *γ*) shows one peak at about 20° and one at about 65°. Within the class of kissing hairpins we often find coaxial stacking, but most of the time only one of the two regular stems stacks with the kissing stem in the middle. With the help of the coarse grained representation we were able to divide the class of kissing hairpins into three different structural families (see Figure 5b-d).

The first family is especially common among (A-)riboswitches. Here the two regular stems are oriented almost parallel ( *γ* below to 32°) with the second one (counting from the 5’ end) stacking onto the kissing stem. The angles *α* and *β* are above 130°. One example is the structure with PDB id 5KPY ^35^ shown in Figure 5b. Here, the link from the first regular stem stacks onto it and interacts with the kissing stem via base multiplets and A-Minor interactions. This way, the roughly parallel orientation of all 3 stems is stabilized by stacking and base-pairing interactions. Interestingly, the kissing stem is more parallel to the first stem than to the second stem, onto which it stacks directly ( *α > β*).

The second structural family is shown in Figure 5c. Here the two regular stems are close to 90°, whereas the kissing stem is along the arc between them. This conformation is found in several groups of non coding RNA including some 23S ribosomal RNA and the cobalamin riboswitch regulatory element (PDB id 4FRN ^36^) shown in Figure 5c.

The two families as well as some conformations in between are observable within intramolecular pseudoknots. As mentioned, forgi is also able to model multiple RNA chains that interact with each other forming an intermolecular complex. One example is the dimerization initiation site of the HIV type 1 (PDB id 1ZCI ^37^), illustrated in Figure 5d and being the only example of the third family of kissing hairpin pseudoknots. Here the kissing hairpin interaction shows a nearly perfect coaxial stacking between all three involved stems. In this case *β* is close to 0°, because stacking takes place at the other side of the kissing stem and thus the complementary angle is close to 180°.

## Discussion

Using the forgi library ^17^ we have analyzed the relative orientation of helices in junctions and pseudoknots. While stacking between adjacent helices is common, we found that their orientation often deviates significantly from co-linearity, suggesting that junctions introduce flexibility into the RNA structure while still maintaining the benefit of stacking interactions. Previous studies that assign coaxial geometries based on manual inspection ^38^ miss this deviation from coaxiality that becomes apparent once you actually calculate the helix axis. Our approach allowed us to perform this analysis on the scale of stems, as opposed to the smaller all-atom scale used by the program DSSR and in previous surveys ^39^ or the level of networks of (noncanonical) base pairs ^38^.

Conversely, we found that out of 1257 pairs of adjacent stems in ribosomal RNA structures with an angle above 140°, only roughly half (608) are annotated as stacking by DSSR. This becomes clear if we consider that the angle between stems is only a proxy for a 5-dimensional orientation parameter. In particular, a large offset means that stem axes with an angle close to 180° can be parallel without being coaxially stacked. Indeed, it is not uncommon that all three stems in a 3-way junction are roughly parallel, with two stems forming a coaxial stack and the third having a higher offset ^40^.

There is growing interest in predicting pseudoknots in RNA structures as they are involved in a variety of biological functions ^41^. The forgi library allows us to easily identify pseudoknots in RNA 3D structures and gather statistics on the frequency of pseudoknot types, sizes, and composition. Kissing hairpins formed between two RNA molecules are known to often form continuous stacks between all three helices, such as the pseudoknot in Figure 5d. In contrast to these intermolecular kissing hairpins, we find that stacking between all three helices is seldom possible in intramolecular pseudoknots. Instead we find that the limited length of all loop segments makes it nearly impossible to form coaxial stacking of all three stems in one line in a single RNA chain. Thus, in the main families of kissing hairpin conformations, the regular (outer) stems are oriented parallelly or almost perpendicular with the kissing helix stacking onto at most one of the two regular stems. These structures are often further stabilized by base triplets like those found in Figure 5b.

Similar to stacking conformations in multi loops, many stacking helices in pseudoknots form angles below 180°. A deviation of the coaxiality between stems can have multiple reasons such as a higher flexibility of short helices within a pseudoknot as well as strain from short stem connections. But there are also structures that show more classical coaxial stacking like PDB id 2XD0, see Figure 5a.

Results and challenges of the application of forgi to the analysis of pseudoknots are described in more detail in the thesis by Beckmann ^42^.

Varying additional parameters like the minimal number of base pairs required to count an interaction as a helix allow for a better understanding about the principles of the formation of three-dimensional RNA structures. The insight in the world of pseudoknots and multi loops presented here can support the improvement of the prediction of RNA structures and help identify unrealistic multi-loop conformations predicted by current RNA structures prediction tools. We are now in the process of implementing additional features for the RNA 3D structure prediction program Ernwin ^20^ based on our findings about stacking and pseudoknots.

## Conclusions

forgi ^17^ is a multi-purpose Python library for dealing with RNA on the levels of sequence, secondary structure and tertiary structure. By providing our code as a Python library, we give users of our tool more flexibility than a single executable program could provide. Furthermore, our code is fully open source, giving researchers the possibility to comprehend and where needed extend the inner workings of the code.

forgi is well documented, with a tutorial available at https://ViennaRNA.github.io/forgi/ and a complete API documentation following the Python standard of docstrings parsable by Sphinx. It contains an extensive automatic test suite (unit and integration tests) and has been optimized for speed, maintainability and usefulness.

## Data availability

### Underlying data

The PDB ids analyzed were taken from version 3.36 of the representative sets of RNA 3D structures ^28^ at a cutoff of 4 Å, http://rna.bgsu.edu/rna3dhub/nrlist/release/3.36/4.0A.

### Extended data

Open Science Framework: 3D based on 2D: forgi 2.0 Extended Data. https://doi.org/10.17605/OSF.IO/HDJRU ^43^. This project contains the following extended data files:

- **assignment_of_classes_to_pdbids.csv**: Assignment of PDB ids to RNA type. These annotations were generated in a semi-manual way.
- **multiloop_angles.csv**: Angles and offsets measured in PDB structures for multi loops, generated with the script describe_rna.py, which is available in the examples folder of forgi.
- **pseudoknot_angles.tsv**: Angles measured in pseudoknots, generated with pseudoknot_analyzer.py, which is available in the examples folder of forgi.

Extended data are available under the terms of the Creative Commons Zero "No rights reserved" data waiver (CC0 1.0 Public domain dedication).

## Software availability

**Software available from:** https://pypi.org/project/forgi/ ( pip install forgi) and Bioconda.

**Archived source code at time of publication:** https://doi.org/10.5281/zenodo.2582870 ^17^.

**License:** GNU General Public License 3.0.

## References

[1] Dawson WK Maciejczyk M Jankowska EJ: Coarse-grained modeling of RNA 3D structure. *Methods.* 2016;103:138–156. 10.1016/j.ymeth.2016.04.026 27125734

[2] Berman H Henrick K Nakamura H: The worldwide Protein Data Bank (wwPDB): ensuring a single, uniform archive of PDB data. *Nucleic Acids Res.* 2007;35(Database issue):D301–D303. 10.1093/nar/gkl971 17142228 PMC1669775

[3] Leontis NB Westhof E: Geometric nomenclature and classification of RNA base pairs. *RNA.* 2001;7(4):499–512. 10.1017/S1355838201002515 11345429 PMC1370104

[4] Gendron P Lemieux S Major F: Quantitative analysis of nucleic acid three-dimensional structures. *J Mol Biol.* 2001;308(5):919–936. 10.1006/jmbi.2001.4626 11352582

[5] Yang H Jossinet F Leontis N: Tools for the automatic identification and classification of RNA base pairs. *Nucleic Acids Res.* 2003;31(13):3450–3460. 10.1093/nar/gkg529 12824344 PMC168936

[6] Sarver M Zirbel CL Stombaugh J: FR3D: finding local and composite recurrent structural motifs in RNA 3D structures. *J Math Biol.* 2008;56(1–2):215–252. 10.1007/s00285-007-0110-x 17694311 PMC2837920

[7] Lu XJ Bussemake HJ Olson WK: DSSR: an integrated software tool for dissecting the spatial structure of RNA. *Nucleic Acids Res.* 2015;43(21):e142. 10.1093/nar/gkv716 26184874 PMC4666379

[8] Zok T Antczak M Zurkowski M: RNApdbee 2.0: multifunctional tool for RNA structure annotation. *Nucleic Acids Res.* 2018;46(W1):W30–W35. 10.1093/nar/gky314 29718468 PMC6031003

[9] Mustoe AM Brooks CL Al-Hashimi HM: Hierarchy of RNA functional dynamics. *Annu Rev Biochem.* 2014;83(1):441–466. 10.1146/annurev-biochem-060713-035524 24606137 PMC4048628

[10] Thiel BC Flamm C Hofacker IL: RNA structure prediction: from 2D to 3D. *Emerg Top Life Sci.* 2017;1(3):275–285. 10.1042/etls20160027 33525808

[11] Lorenz R Bernhart SH Höner Zu Siederdissen C: ViennaRNA Package 2.0. *Algorithms Mol Biol.* 2011;6(1):26. 10.1186/1748-7188-6-26 22115189 PMC3319429

[12] Cock PJ Antao T Chang JT: Biopython: freely available Python tools for computational molecular biology and bioinformatics. *Bioinformatics.* 2009;25(11):1422–1423. 10.1093/bioinformatics/btp163 19304878 PMC2682512

[13] Hamelryck T Manderick B: PDB file parser and structure class implemented in Python. *Bioinformatics.* 2003;19(17):2308–2310. 10.1093/bioinformatics/btg299 14630660

[14] Knight R Maxwell P Birmingham A: PyCogent: a toolkit for making sense from sequence. *Genome Biol.* 2007;8(8):R171. 10.1186/gb-2007-8-8-r171 17708774 PMC2375001

[15] Smit S Rother K Heringa J: From knotted to nested RNA structures: a variety of computational methods for pseudoknot removal. *RNA.* 2008;14(3):410–416. 10.1261/rna.881308 18230758 PMC2248259

[16] Rother M Rother K Puton T: ModeRNA: a tool for comparative modeling of RNA 3D structure. *Nucleic Acids Res.* 2011;39(10):4007–4022. 10.1093/nar/gkq1320 21300639 PMC3105415

[17] Thiel B Kerpedjiev P tcarlile: Viennarna/forgi: Forgi version 2.0.2019 10.5281/zenodo.2582870

[18] Licht K Jantsch MF: Rapid and dynamic transcriptome regulation by RNA editing and RNA modifications. *J Cell Biol.* 2016;213(1):15–22. 10.1083/jcb.201511041 27044895 PMC4828693

[19] Dimitropoulos D Ionides J Henrick K: Using MSDchem to search the PDB ligand dictionary. *Curr Protoc Bioinformatics.* 2006; Chapter 14: Unit14.3. 10.1002/0471250953.bi1403s15 18428761

[20] Kerpedjiev P Höner Zu Siederdissen C Hofacker IL: Predicting RNA 3D structure using a coarse-grain helix-centered model. *RNA.* 2015;21(6):1110–1121. 10.1261/rna.047522.114 25904133 PMC4436664

[21] Schlick T: Adventures with RNA graphs. *Methods.* 2018;143:16–33. 10.1016/j.ymeth.2018.03.009 29621619 PMC6051918

[22] Steffen P Voss B Rehmsmeier M: RNAshapes: an integrated RNA analysis package based on abstract shapes. *Bioinformatics.* 2006;22(4):500–503. 10.1093/bioinformatics/btk010 16357029

[23] Oliphant TE: Guide to NumPy. 2nd Edition. CreateSpace Independent Publishing Platform,2015 Reference Source

[24] Jones E Oliphant T Peterson T: SciPy: Open source scientific tools for Python, since 2001. [online, accessed 8 Oct 2018]. Reference Source

[25] Hagberg AA Schult DA Swart PJ: Exploring network structure, dynamics, and function using networkx. In Gaël Varoquaux, Travis Vaught, and Jarrod Millman, editors, *Proceedings of the 7th Python in Science Conference* Pasadena, CA USA.2008;11–15. Reference Source

[26] McKinney W: Data structures for statistical computing in python. In Stéfan van der Walt and Jarrod Millman, editors, *Proceedings of the 9th Python in Science Conference* 2010;51–56. Reference Source

[27] McKinney W: pandas: a foundational python library for data analysis and statistics. *Python for High Performance and Scientific Computing* 2011;1–9. Reference Source

[28] Leontis NB Zirbel CL: Nonredundant 3D structure datasets for RNA knowledge extraction and benchmarking. In *Nucleic Acids and Molecular Biology* Springer Berlin Heidelberg.2012;281–298. 10.1007/978-3-642-25740-7_13

[29] Tyagi R Mathews DH: Predicting helical coaxial stacking in RNA multibranch loops. *RNA.* 2007;13(7):939–951. 10.1261/rna.305307 17507661 PMC1894924

[30] Hunter JD: Matplotlib: A 2D graphics environment. *Comput Sci Eng.* 2007;9(3):90–95. 10.1109/mcse.2007.55

[31] Reidys CM Huang FW Andersen JE: Topology and prediction of RNA pseudoknots. *Bioinformatics.* 2011;27(8):1076–1085. 10.1093/bioinformatics/btr090 21335320

[32] Suzuki T Nakamura A Kato K: Structure of the *Pseudomonas aeruginosa* transamidosome reveals unique aspects of bacterial tRNA-dependent asparagine biosynthesis. *Proc Natl Acad Sci U S A.* 2015;112(2):382–387. 10.1073/pnas.1423314112 25548166 PMC4299244

[33] Schrödinger, LLC: The PyMOL molecular graphics system.version 1.8.2015.

[34] Blower TR Pei XY Short FL: A processed noncoding RNA regulates an altruistic bacterial antiviral system. *Nat Struct Mol Biol.* 2011;18(2):185–191. 10.1038/nsmb.1981 21240270 PMC4612426

[35] Porter EB Polaski JT Morck MM: Recurrent RNA motifs as scaffolds for genetically encodable small-molecule biosensors. *Nat Chem Biol.* 2017;13(3):295–301. 10.1038/nchembio.2278 28092358 PMC5310984

[36] Johnson JE Jr Reyes FE Polaski JT: B _12_ cofactors directly stabilize an mRNA regulatory switch *Nature.* 2012;492(7427):133–137. 10.1038/nature11607 23064232 PMC3518761

[37] Ennifar E Dumas P: Polymorphism of bulged-out residues in HIV-1 RNA DIS kissing complex and structure comparison with solution studies. *J Mol Biol.* 2006;356(3):771–782. 10.1016/j.jmb.2005.12.022 16403527

[38] Laing C Schlick T: Analysis of four-way junctions in RNA structures. *J Mol Biol.* 2009;390(3):547–559. 10.1016/j.jmb.2009.04.084 19445952 PMC2777522

[39] Holbrook SR: Structural principles from large RNAs. *Annu Rev Biophys.* 2008;37(1):445–464. 10.1146/annurev.biophys.36.040306.132755 18573090

[40] Lescoute A Westhof E: Topology of three-way junctions in folded RNAs. *RNA.* 2006;12(1):83–93. 10.1261/rna.2208106 16373494 PMC1370888

[41] Staple DW Butcher SE: Pseudoknots: RNA structures with diverse functions. *PLoS Biol.* 2005;3(6):e213. 10.1371/journal.pbio.0030213 15941360 PMC1149493

[42] Beckmann IK: Identification and Classification of Pseudoknots and their Impact on RNA 3D Structure Prediction. Master’s thesis, University of Vienna,2018 Reference Source

[43] Thiel BC Beckmann IK Kerpedjiev P: 3D based on 2D: Forgi 2.0 Extended Data.2019 10.17605/OSF.IO/HDJRU PMC6480952 31069053

