# Supplementary material for: 3D based on 2D: Calculating helix angles and stacking patterns using forgi 2.0, an RNA Python library centered on secondary structure elements
Source: F1000Res. 2019 Apr 23;8:ISCB Comm J-287. Originally published 2019 Mar 14. [Version 2] doi: 10.12688/f1000research.18458.2 (PMC6480952; doi:10.12688/f1000research.18458.2)
Supplement: Peer review history

# Peer review: Reviewer response for version 2

**DOI:** 10.5256/f1000research.20631.r47470

**Published:** 2019-04-23

**Authors:** Marta Szachniuk

**Affiliations:** 1 Institute of Computing Science & European Centre for Bioinformatics and Genomics, Poznan University of Technology, Poznań, Poland

I'm satisfied with all modifications the authors made.

I have read this submission. I believe that I have an appropriate level of expertise to confirm that it is of an acceptable scientific standard.

# Peer review: Reviewer response for version 1

**DOI:** 10.5256/f1000research.20195.r45731

**Published:** 2019-03-27

**Authors:** Jerome Waldispühl, Roman Sarrazin-Gendron

**Affiliations:** 1 School of Computer Science, McGill University, Montreal, QC, Canada

The authors present a new version of the forgi software, a python library to analyze the tertiary structure of secondary structure elements in RNA. It takes as input almost any format of 2D or 3D information.

 This manuscript describes a new method for easily obtaining the angles and stacking patterns in a multi-branched loop and a pseudoknot/kissing hairpin. The authors validate their results by testing the software on known pseudo-knots and junctions, and obtain results that are consistent with the state-of-the-art knowledge of RNA local structure organization.

 The authors highlight significant improvements compared to previously published packages and specifically all-atom based methods. For instance, a deviation from coaxiality is very common but can be easily missed if a manual inspection is performed without doing computations.

 The software is clearly presented, well-validated, freely available and open source. The results presented are significant and the tool will be very useful to the RNA bioinformatics community.

 Although, this manuscript does not discuss extensively the analysis of the results, the latter is included in the first author’s thesis, and freely accessible as further reading.

 `

**Minor remarks**

- Page 4: ‘’with and without missing residues’’, it is easy to imagine how the graph without the missing residue is, but how are the ‘’missing residues’’ added? (i.e. are you inferring which residues are missing, and if yes, how?)
- Page 5: The authors mention a ‘’cleaned version’’ of the PDB. Is the only ‘’cleaning’’ performed is the conversion of the residue names?
- Page 5: It would be helpful to clarify what is a ‘’simplified example’’ of the code? What sort of simplification was applied to it? Is the real code significantly more complicated to use than the example? If it is not, the authors might want to consider rewording this as it appears to undermine the real simplicity of use of forgi (which is very user friendly).

We have read this submission. We believe that we have an appropriate level of expertise to confirm that it is of an acceptable scientific standard.

# Response

**Published:** 2019-04-01

**Authors:** Bernhard Thiel

**Affiliations:** University of Vienna, Austria

Thank you for the constructive comments.

Our PDB and CIF parsers extract the information about missing residues from the files. For this purpose we have patched the biopython PDB parsing routine; this has already been merged into the biopython source. Following your input, we have revised the text to reflect this.

Yes, the cleaned version only has modified residues renamed and non-rna molecules removed. We hope this is now clear from the revised text.

We have expanded the sample code to compute the proper direction of helices, thus it is now fully functional and no longer "simplified".

We have also added the code used to calculate the offset.

# Peer review: Reviewer response for version 1

**DOI:** 10.5256/f1000research.20195.r45753

**Published:** 2019-03-27

**Authors:** Marta Szachniuk

**Affiliations:** 1 Institute of Computing Science & European Centre for Bioinformatics and Genomics, Poznan University of Technology, Poznań, Poland

The article is written in a clear and concise way. Illustrations perfectly complement the content. I have only 3 comments to the authors and I would like them to be taken into account in the revision:

1. For unknown reasons, the authors introduced a new spelling of the name pseudoknot. Throughout the whole article, they use spelling with space: "pseudo knots". I haven't met such a form before. The authors of the article also used the term "pseudoknot" and not "pseudo knot" in their previous works. So, the spelling should be corrected.
2. On page 3, the authors write about FR3D suite, but they do not cite the paper about the tool. Please, include the appropriate citation here.
3. As the paper subject is calculating helix angles and stacking parameters, I would expect to see the formulas used to calculate these data (or - at least - the pseudocode showing how they are calculated). The lack of such details is serious negligence.

I have read this submission. I believe that I have an appropriate level of expertise to confirm that it is of an acceptable scientific standard, however I have significant reservations, as outlined above.

# Response

**Published:** 2019-04-01

**Authors:** Bernhard Thiel

**Affiliations:** University of Vienna, Austria

Thank you for the constructive comments.

We have now included the exact formulas for the helix angles as requested. In particular this makes clear what sign we use for the helix vectors.

The FR3D reference was present in our LaTeX source and the resulting HTML version, but got lost in the pdf conversion. This has been fixed in the revised version.
